# Fathers’ engagement in a parenting program primarily intended for female caregivers: An early qualitative process evaluation in Western Kenya

**DOI:** 10.1371/journal.pgph.0003520

**Published:** 2024-10-11

**Authors:** Joshua Jeong, Juliet K. McCann, Alina Bhojani, Zane Maguet, Malia Uyehara, Michael Ochieng

**Affiliations:** 1 Hubert Department of Global Health, Rollins School of Public Health, Emory University, Atlanta, Georgia, United States of America; 2 B&M Consult, Nairobi, Kenya; Akademia Wychowania Fizycznego Jozefa Pilsudskego w Warszawie, POLAND

## Abstract

Parenting programs predominantly target one caregiver of the child or most commonly the child’s mother. However, fathers are also important caregivers whose engagement in interventions can benefit child health, nutrition, and development. In August 2023, a qualitative process evaluation was conducted during the first quarter of implementation to assess initial fidelity, quality, and outcomes of a parenting program in rural Western Kenya. In-depth interviews were conducted with female and male caregivers along with in-depth interviews and focus group discussions with program delivery agents. This secondary analysis specifically focused on stakeholders’ perceptions of father involvement in the program and aimed to identify barriers and facilitators to fathers’ participation, initial program impacts when fathers were involved, and recommendations for increasing father inclusion. Thematic content analysis was conducted, and data were triangulated across stakeholder groups. Overall, relatively few fathers participated in the program. Nevertheless, for the rare cases of participating fathers, stakeholders highlighted positive changes in fathers’ caregiving attitudes and practices. Key barriers to fathers’ program engagement included restrictive gender norms and perceived opportunity costs. Stakeholders suggested several strategies for better reaching fathers, including providing financial incentives and flexible scheduling of sessions. Overall, we found that fathers’ participation and program experiences were starkly different from those of mothers. Gender-responsive program adaptations and father-targeted implementation strategies are likely to increase the fathers’ engagement in parenting programs, which in turn may facilitate greater program impacts on family caregiving and child outcomes. Future evaluations of parenting programs should combine qualitative and quantitative approaches to more comprehensively assess program impacts on fathers and over time.

## Introduction

Millions of young children in low- and middle-income countries (LMICs) are failing to reach their full developmental potential [1]. Parenting interventions are one successful approach for improving early child development (ECD) globally [2]. While various types of parenting programs have been implemented in LMICs (e.g., home visiting, community group sessions, integration at pediatric clinics), the vast majority have focused primarily on mothers–with few reaching fathers [3]. For example, in a global systematic review of 102 parenting programs, only 7% of interventions invited fathers as participants [4].

Nevertheless, over the past decade, there has been increased momentum around engaging fathers in parenting programs and other types of caregiving programs more generally in LMICs. A recent systematic review found that father-inclusive interventions can achieve a wide range of benefits, including improved maternal and paternal caregiving practices, couples’ relationship dynamics, and child development outcomes [5]. Fatherhood programs also have the potential to address harmful gender norms and redress power imbalances between men and women to improve gender equality within and across families and communities [6, 7]. For instance, while the primary role of fathers in many settings across LMICs including Western Kenya are traditionally centered around financially providing for the family and with mothers mainly responsible for childcare activities (e.g., playing, feeding, bathing) and household chores, gender-transformative parenting interventions can increase father involvement in childcare and household chores and result in more equitable distributions of caregiving practices [8, 9]. Moreover, studies across LMICs have demonstrated how father involvement in stimulation activities is associated with improved ECD outcomes [10, 11].

While father-inclusive interventions can have a wide range of benefits, a key factor underlying their success is implementation fidelity and quality [3]. Previous studies have highlighted various implementation-related challenges with respect to engaging fathers in parenting programs. For example, multiple programs have struggled with relatively low participation rates of fathers compared to mothers due to various interconnected factors such as poverty, restrictive gender norms, and men’s time use patterns [3, 12]. Relating to fathers’ attendance, other studies have noted how fathers have unique preferences for the timing, format, and structure of parenting sessions that are often not considered in the program design or implementation strategy [13, 14].

To date there are relatively few studies that have examined the implementation experiences and community perceptions around father involvement in parenting programs in LMICs. One notable exception is a qualitative process evaluation of the ‘Parenting for Respectability’ program, which is a parenting program for mothers and fathers with children 0–17 years of age in Uganda. This study found that although fathers were eager to improve their relationships with their children and wives through the program, restrictive gender norms and social stigma around male engagement in childcare activities were major barriers [15]. Another study in Malawi evaluated mothers’ perceptions of father engagement in a clinic-based parenting program for children under 2 years [16]. Most mothers reported that fathers had favorable views about the parenting program, with some fathers becoming more involved with their young child at home. At the same time, the majority of mothers believed fathers were not comfortable participating in the program because of social norms and the belief that parenting sessions are for only women as well as time-related barriers. Together these initial studies highlight the crucial need to investigate fathers’ experiences and perceptions and identify the contextual determinants influencing men’s engagement in parenting programs across LMICs.

The objective of this study was to explore fathers’ engagement in the Moments that Matter (MTM) parenting program in Western Kenya. MTM is a community-based parenting program for caregivers with children under 3 years of age that uses peer group sessions and home visits to promote nurturing care and ultimately improve ECD [17]. We designed a qualitative process evaluation of the MTM program and conducted in-depth interviews and focus groups discussions with various program stakeholders as well as direct observations of sessions. For this analysis, we explored the extent to which male caregivers engaged in the program, the perceived barriers and facilitators to men’s participation, and stakeholders’ recommendations for enhancing father involvement in the program. By understanding fathers’ program engagement from the perspectives of various program stakeholders, we aim to inform gender-responsive program adaptations to more effectively reach and engage both female and male caregivers and ultimately better enhance parenting and ECD outcomes.

## Methods

### Study design

Data for this study were collected as part of a larger qualitative process evaluation that was conducted four months into implementation of the ongoing MTM parenting program. The main goal of this process evaluation was to assess overall fidelity, acceptability, and initial effectiveness of the MTM program [18]. For this secondary analysis, we specifically explored fathers’ experiences participating in the MTM program, the initial impacts of the program on father-related outcomes, and recommendations to increase the future engagement of fathers in MTM. We analyzed data from in-depth interviews (IDIs) and focus group discussions (FGDs) with relevant stakeholder groups (e.g., participating male and female caregivers, MTM delivery agents, program staff) and notes taken from semi-structured observations of MTM group sessions and home visits.

### MTM program description

MTM is a community-based, multi-component parenting intervention being implemented in in Western Kenya across Borabu sub-county in Nyamira county and Luanda sub-county in Vihiga county. Implementation of MTM is led by the Anglican Church of Kenya Development Services (ADS)-Nyanza (Nyamira) and ADS-Western (Vihiga) with program oversight and technical support from Episcopal Relief & Development. The aim of MTM is to promote nurturing care and positive social and behavioral change by building caregiver capacities and mobilizing agents of change within the community. While MTM enrolls and mainly focuses on the primary caregivers of children aged 0 to 3 years (typically mothers), other caregivers including fathers are also invited to attend sessions.

The MTM program is being delivered by ECD Promoters and faith leaders over an 18-month implementation period for a total of 36 program contacts (18 monthly group sessions with up to thirteen primary caregivers and 18 monthly home visits). ECD Promoters are volunteers who were recruited and trained specifically for this program. Faith leaders, in contrast, have existing roles within their communities that are being leveraged for the purposes of this program.

During group sessions, ECD Promoters use a structured curriculum to counsel caregivers on topics about parenting (e.g., responsive care, early learning), child health and nutrition, and livelihood training. While most sessions aim to enhance caregiving broadly, fathers’ parenting is specifically addressed in meetings on caregiving gender roles and family violence. Father involvement is also mentioned at the end of every group session when participating caregivers are asked to assign a family member to practice an action from the session, again with a particular emphasis on fathers. Finally, in addition to the group sessions, fathers are encouraged to participate in monthly home visits especially among mothers who live with the child’s father.

### Sampling

This study was conducted in 12 purposively selected villages receiving the MTM program. Within both Nyamira and Vihiga county, we randomly selected two high performing and two low performing sites based on average caregiver attendance at the group sessions, with two additional sites randomly selected in each county for home visit observations. In each of these selected villages, we invited the ECD Promoter to participate in an IDI (12 IDIs). IDIs were also carried out with a random selection of faith leaders (8 IDIs), female caregivers (15 IDIs), and male caregivers (5 IDIs). Additionally, in each site we conducted structured observations of either the group sessions (7 observations) or the home visits (5 observations). Finally, for the FGDs, faith leaders, and ECD Promoters working in nearby villages were invited to convene for a group discussion. One FGD with ECD Promotors and one FGD with faith leaders was conducted in each county (4 FGDs), with approximately 8 participants per group. Finally, we conducted one IDI with an ADS field staff member in each county.

### Data collection

We developed semi-structured topic guides for each data collection method (IDIs and FGDs) and stakeholder group (e.g., female caregivers, male caregivers, ECD Promoters, faith leaders, and program staff). The guides included questions on perceived program fidelity, satisfaction, and initial effectiveness, with probes incorporated throughout to identify barriers and facilitators. For example, fathers themselves were asked about the facilitators and barriers to their program participation (e.g., “How easy or difficult has it been for you/fathers to attend the sessions?”) and their experiences with and attitudes towards the program (e.g., “How do you feel about there being more women than men in this program?”). Other stakeholder groups, such as female caregivers, delivery agents, and program staff, were asked questions about their interactions with fathers in the program (e.g., “To what extent do you interact and meet with fathers through your role as a faith leader in the MTM program?”) and their perceptions of fathers’ experiences (e.g., “What makes it easy or difficult for fathers to be engaged in the MTM program?”). Finally, all respondents were asked about suggested improvements to the program specifically in terms of increasing fathers’ participation (e.g., “What can be done to get more male caregivers (fathers) engaged in this program?”). Prior to data collection, topic guides were piloted in another MTM community and iteratively improved for clarity and scope.

Data were collected in August, 2023 by a team of four Kenyan research assistants under the guidance of one field supervisor. All research assistants were fluent in Kiswahili and English, held bachelor’s degrees, and had prior experience with qualitative research. Research assistants received a 4-day training that included classroom instruction (i.e., qualitative research methodologies, interviewing skills, study overview, and topic guides) followed by supervised field practice in a non-study community. Informed consent was obtained by all participants prior to each interview and discussion, which were carried out in a private location in the community or the respondent’s home. All interviews and discussions were conducted in Kiswahili. Audio recordings of these interviews and discussions were then translated and transcribed into English transcripts, which were then independently reviewed by the research field supervisor for quality assurance.

### Data analysis

A team of trained researchers analyzed the data using thematic content analysis. An initial codebook for the IDIs and FGDs were developed based on the topic guide and study framework. These codes were then piloted on a subset of transcripts and iteratively modified based on group discussions. Five research analysts coded the transcripts using Atlas.ti. Approximately half of the transcripts were reviewed by a second analyst to ensure quality and consistency in team coding. Additionally, the team engaged in weekly meetings to review any coding discrepancies, revise the codebook, and discuss emerging themes.

For this secondary analysis, we specifically explored emerging themes related to fathers’ experiences participating in the MTM program, the impacts of the program on fathers and their behaviors, and suggestions on how to improve the engagement of fathers in the program. Themes were identified through a process of systematically synthesizing codes relevant to these topic areas, summarizing findings, and discussing these results with the study team. Additionally, we triangulated findings across various stakeholder groups to examine convergence of results.

### Ethical approval

This study received ethical approvals from the Harvard T.H. Chan School of Public Health and the Jaramogi Oginga Odinga Teaching and Referral Hospital. A research license was also obtained for this study from the National Commission for Science, Technology, and Innovation in Kenya. Written informed consent was obtained from all study participants.

## Results

In total, the sample included 67 participants. The average age of the sampled male caregivers was 48.4 years, whereas the female caregivers were 33.1 years of age. The majority of caregivers were the child’s biological parent (80% of male caregivers were child’s father and 87% of female caregivers were child’s mother). Male caregivers were more educated than female caregivers with 80% versus 60% respectively completing secondary school or higher. Agriculture was the primary occupation among male caregivers (60%), whereas female caregivers engaged in various jobs, including agriculture (27%) and small business (20%). At the time of the study, index children were on average 17 months of age, which ranged from 5 to 32 months. Most of the interviewed ECD Promoters (88%) and faith leaders (62%) were female, and both ADS supervisors were female.

### Fathers’ program participation

Across IDIs and FGDs, stakeholders generally agreed that male caregivers were much less engaged in the program compared to female caregivers. Stakeholders unanimously equated the phrase “male caregiver” to refer to fathers. While many stakeholders reported that small numbers of male caregivers were sometimes present at group sessions, female caregivers always outnumbered male caregivers by a significant margin. For example, one ECD Promoter shared, “*When it comes to fathers*, *we sometimes have two or one or none at all in our meetings*. *Because they say they go to look for money*, *because if they both attend*, *they may have nothing to take home for food*.*” (ECD Promoter*, *IDI #1*, *Vihiga)* Of the seven observed group sessions, two had no male caregivers at all in attendance and two more had only one male caregiver. The remaining three sessions each had five or fewer male caregivers present. Some stakeholders expressed the belief that male caregivers were more likely to participate in home visits than group sessions, with one female caregiver sharing, “*I like home visits because when [the ECD promoter] visits me at home sometimes I am with my husband so he gets to learn something from the session and he applies the same*, *the home visits are good because they involve both parents because as you know men don’t like attending these sessions*.*”* (*Female Caregiver*, *IDI #1*, *Vihiga*) However, male caregiver attendance was still relatively low even at home visits. None of the five observed home visits had a male caregiver present.

### Barriers

We identified four main barriers to male caregiver participation in the program. These barriers were: 1) perceived lack of time due to men’s roles as breadwinners for the family, 2) restrictive gender norms and expectations about childcare as a maternal and not paternal responsibility, 3) fathers’ alcohol use, and 4) men’s doubts about the usefulness of the program.

The most frequently mentioned barrier to male caregiver involvement in the program was time constraints due to father’s responsibility to financially provide for the family. Men and women equally valued fathers’ income-generating activities and considered paternal employment as the primary role of fathers. For example, one father shared, *“As fathers our spouses expect us to bring food every evening*, *because of the responsibility*, *it becomes difficult to attend all the meetings*.*” (Father*, *IDI #1*, *Vihiga)* Several individuals shared how even though some fathers wanted to attend group sessions, they could not afford to miss work and opportunities to earn money. When asked whether her partner attends any of the program sessions, one mother responded, *“No*. *He really wants to attend when I tell him about all the knowledge that we are getting but he has to go to work*. *Like today*, *he wanted to attend but got a call from his boss and he had to get to work unfortunately so that he can also be able to provide food for us*.*" (Mother*, *IDI #1*, *Nyamira)*.

Intertwined with the cultural norm of fathers as providers, restrictive gender norms were another major barrier to fathers’ engagement in the program. Respondents–especially ECD Promoters and faith leaders–reiterated how parenting activities and the day-to-day care of young children were considered maternal and not paternal responsibilities. For example, one ECD Promoter shared, *“Accepting [the program] for fathers is difficult because they feel that the training concerns mothers and not fathers*. *They say that their responsibility is to provide for the family and not to look after the child*, *which to them is a mother’s responsibility*.*" (ECD Promoter*, *IDI #2*, *Vihiga)* Similarly, one faith leader explained how many fathers are reluctant to participate because of these gender norms by saying, *"The fathers are difficult to deal with*, *they are usually asking why they should attend the training*, *when the children belong to the mother… They take parenthood as a role of the wives*, *not them*. *They think someone will do it for them*.*" (Faith Leader*, *IDI #1*, *Nyamira)* It is worth noting that many female caregivers also endorsed these restrictive gender norms themselves by not expecting male caregivers to attend or justifying fathers’ employment as more important than participating in the program. For example, one mother explained, *"Many fathers are trying to make ends meet as they are casual laborers*, *we can’t have all of us attending and leave work*. *So*, *it gets hard for many of the fathers to attend and leave work… hence you will find many women*.*" (Female Caregiver*, *IDI #7*, *Nyamira)*.

Although not as frequently mentioned as the previous barriers, another commonly identified barrier to father engagement with the program was men’s frequent alcohol use. Male caregivers, female caregivers, and delivery agents alike acknowledged that many male caregivers struggle with alcohol use and that it negatively impacts their ability to engage with the program. One female caregiver commented on how pervasive alcohol abuse is in her community and how much it can inhibit group session participation by saying, *"Most fathers and male caregivers do not attend because they are mostly drunk*. *Our area is affected by alcohol abuse and most men have succumbed to the vice*.*" (Mother*, *IDI #7*, *Vihiga)* A faith leader pointed out how alcohol can also present a barrier to home visits, saying, *"Men don’t like to be visited regularly by the promoters at their homes every time*, *because some of them are drunkards*, *so I have told the promoters to be aware of that*.*" (Faith Leader*, *IDI #2*, *Nyamira)*.

Finally, several stakeholders mentioned fathers’ negative attitudes towards the program, such as it being unhelpful and a waste of time not only for themselves but for their wives as well. While this was shared by all stakeholders, female caregivers most frequently expressed this idea that their husbands were skeptical and lacked awareness about the importance of the program. One mother shared that fathers often do not see the value in the program, saying, *"You know the fathers despise the training because they haven’t seen anything beneficial*.*" (Mother*, *IDI #3*, *Nyamira)* Another mother expressed the same sentiment by commenting, *"[Fathers and other male caregivers] don’t believe in the training as they tend to think that we are just wasting time and even think that the training is a hoax*.*" (Mother*, *IDI #7*, *Nyamira)*.

### Facilitators

We identified four main facilitators of father involvement in the program. These facilitators were: 1) understanding of the program goals, 2) delivery agent encouragement, 3) strategic scheduling of sessions, and 4) family and peer encouragement.

In contrast to the previously noted barrier of fathers’ skeptical attitudes towards the program, it was noted by several individuals that the fathers who attended a session or two often understood the goals of the program. Once fathers had an initial experience with the program, they were more willing to return for subsequent sessions. One ECD Promoter shared, *“We have success in few fathers who understand the program*. *They like the session lessons*. *For example*, *there is one who told me that if it were not for work*, *he would be attending every meeting*. *He also promised that he will try to plan himself to be attending the meetings… He is very willing*.*" (ECD Promoter*, *IDI #1*, *Vihiga)* A faith leader added that it was the male caregivers who attended a session that began to understand why it was significant by sharing, *"First*, *fathers said this program is for women*. *The one who attended the training was able to realize the importance of the training and learned that raising the child is not a mother’s responsibility*, *but all parents should take part*.*" (Faith Leader*, *IDI #1*, *Vihiga)*.

Stakeholders also shared that the encouragement and support of delivery agents—ECD promoters and faith leaders—increased male caregiver engagement in the program. For example, one father mentioned his ECD Promoter in helping him understand and accept the program’s significance, saying, *"Many things made me take part in the program*. *An ECD Promoter explained to me about the program and how as men we are supposed to come in and support our homes in raising our children*. *It expanded my knowledge on how to interact with my family and raise my children*.*" (Father*, *IDI #1*, *Vihiga)* Several delivery agents of both types (ECD Promoters and faith leaders) expressed the idea that faith leaders had the ability to be especially influential on men and their interest in the program. For example, an ECD Promoter said:

"*What also makes it easy for [male caregivers] to come is when we go with the faith leaders and our supervisors… to the group session. When they see them there then they see the importance of this program. We just encourage them to attend when they are available, and they accept depending on their availability. Even when I get an issue in a home visit, I also inform the faith leader and my supervisor and then we visit that home. When the male caregivers see us together, they become more receptive and start attending the following learning session. They even know when to attend, the group learning sessions together with the mothers." (ECD Promoter, IDI #3, Nyamira)*

A faith leader supported the idea that they had more sway with male caregivers by saying, *“Some [fathers] are really following our teachings and when you do home visits you see their involvement*. *The more we keep encouraging them*, *the more they keep changing for better in family matters like proper raising of children*.*" (Faith Leader*, *IDI #4*, *Vihiga)*.

Additionally, delivery agents emphasized the importance of accommodating fathers’ schedules and meeting them where they are most available. They highlighted how planning visits at times that were most convenient for not only the mother but also the father increased the likelihood of fathers’ program participation. An ADS supervisor stated, *"We encourage the promoter to request for the time that both the father and mother are home and to plan such a visit earlier*. *That way*, *it is easier for the fathers who have accepted and have understood what the project entails to create time*. *We are working with their availability*, *and we avoid interfering with their other activities productive time*.*"* (ADS Supervisor, IDI #2) Similarly, an ECD promoter highlighted the significance of timing, the success of scheduling home visits when fathers return from work, and collaborating with mothers to identify suitable times for fathers to participate in sessions.

Finally, male caregivers’ positive relationships with their partners, children, and peers also facilitated program participation. The influence of close relationships was manifested in several ways, including direct encouragement from partners, male caregivers’ own desire to be good fathers, and support from other fathers. One father pointed out the important role his spouse had played in facilitating his engagement with the program, saying, *“My wife has always pushed me to go and listen to what is going on*. *She always reminds me when I am home and there is a meeting*. *I also want to learn more on how to raise my child because it is a responsibility of every father to raise their child well*, *so I have the urge because I want to raise my child well*, *better than others*.*” (Father*, *IDI #1*, *Vihiga)* A mother added, *"For those male caregivers coming here they have children*, *it’s through their wives that they started attending the sessions*, *maybe the wives applied what they learnt which motivated them to come to learn*. *If they could not have done well*, *they would not have come*.*" (Mother*, *IDI #5*, *Nyamira)* Additionally, male caregivers began acting as champions among their peers to encourage others’ participation. For example, one ECD Promoter shared, *"In the beginning*, *it was hard but as we continued*, *some male caregivers had started helping me to tell the other male caregivers from the group meetings*.*" (ECD Promoter*, *IDI #2*, *Nyamira)*.

### Program impacts on male caregivers

Even though relatively few male caregivers participated in the parenting program, all respondents described some positive impact the program was having on fathers. These benefits were reported by delivery agents, female caregivers, and fathers themselves. Respondents shared three domains of improvements among fathers. First, there were improvements in fathers’ knowledge and practices about nurturing care, including topics like parenting, nutrition, and child health. Through home visits, delivery agents further confirmed that some fathers enacted positive parenting practices with their children, through holding and playing with their children. Second, respondents highlighted improvements in couples’ relationship dynamics and the overall family caregiving environment. Finally, respondents noted positive changes in men’s attitudes towards parenting, gender equality, and participation in the program specifically. Stakeholders mentioned no negative impacts of the program on fathers.

The positive impacts of the program were most often discussed in terms of improvements in fathers’ caregiving behaviors with young children. Stakeholders reported that male participants became more willing to engage in parenting and activities pertaining to early childhood development. Additionally, it was often pointed out that fathers were gaining new knowledge about the importance of balanced child nutrition. One mother shared about her husband, *“He never used to like seeing children playing near him but nowadays he plays with them*, *at times you even see him feeding the children and he does it so well*.*” (Mother*, *IDI #2*, *Vihiga)* This point is further supported by a father who described how he learned the importance of dedicating time to play with his children, which he had not been aware of previously. Fathers were also reported as learning about other nurturing care topics such as good hygiene practices and caring for children’s health. Encapsulating several important elements of fathers’ caregiving, one ECD Promoter commented, *“There have been some positive changes because they have learnt about caring for their children*. *The fathers are able to stay with their children and cook for them and change their dirty clothes*. *They have learnt how to discipline their children*. *The caregivers have started having a good relationship with their partners and being involved in their children’s life*.*” (ECD Promoter*, *IDI #4*, *Nyamira)* Another ECD Promoter highlighted how she witnessed a father newly taking his child to the clinic and other childcare activities that are traditionally viewed as maternal responsibilities.

In addition to positive changes between fathers and children, stakeholders highlighted the positive impacts of the program on couples’ relationships and the overall family caregiving environment. Several male caregivers commented on the improvement in their relationship with their wives. One father emphasized the decreased conflict between him and his wife since joining the program by saying, *“…We have never quarreled at home with my wife since I got into the program*, *we talk about love first which is making us to be understanding*,*” (Father*, *IDI #3*, *Nyamira)* which was further supported by an observation from an ECD Promoter who shared about the reduced quarreling he heard within households. A male caregiver similarly highlighted becoming more involved in childcare activities and sharing these responsibilities more with his partner, *“I used to quarrel*, *never helped my wife in taking care of the child*. *But now it’s different*. *We help each other to take care of the child*. *When my wife goes to the market*, *I stay with the child until she comes back*.*” (Male caregiver*, *IDI #1*, *Nyamira)* Male caregivers also expressed the benefits and value they perceived from participating in the program, both for themselves and their families. One father described, *“[The program] has encouraged me to stay with my children*. *This program has instilled good behaviors in me*, *and I now behave well to my family because they are still young*. *When they are wrong*, *I correct them well without violence*. *I am now very humble to my children*.*” (Father*, *IDI #2*, *Vihiga)*.

Finally, various stakeholders noted improvements in fathers’ attitudes, including with respect to men’s roles in childcare and perceptions about the value of parenting programs and MTM specifically. For example, one father shared, *“Through this program*, *I realized that when I play with the child she enjoys so much*. *When I am home*, *we play in the soil using different toys*. *In this program*, *I have learnt that being close to your child should be done by all parents not only mothers*, *and even fathers should be there for the children and not just going for work*.*” (Father*, *IDI #1*, *Vihiga)* One ECD Promoter commented on the positive impacts of the parenting program in challenging social norms that fathers who took part in parenting were a “weakling” by explaining, *“Because of the training*, *people have now embraced living in harmony*, *but before*, *everyone in the house would do their separate duties without regard for each other*. *For example*, *now if the mother goes for other errands*, *the father takes over the baby*. *At least now they have started to help one another*. *This is because earlier*, *if you saw a father taking care of the baby*, *people would conclude that he’s a weakling*. *But now*, *with the program*, *fathers have embraced caring for the baby*.*” (ECD Promoter*, *IDI #1*, *Vihiga)*.

### Recommendations for increasing fathers’ program engagement

All stakeholders recommended providing incentives to increase father participation in the program. First, respondents noted that male caregivers prioritize income-generating activities, and therefore providing monetary compensation would increase men’s participation. One mother noted, *“For men it’s all about money*, *if they can be given 100 and 200 Kenyan shillings [~$1–2 USD] when they attend the session*, *they will come out in big numbers*.*”* (*Mother*, *IDI #6*, *Nyamira*) Recommendations for monetary incentives ranged from 50 to 300 Kenya shillings per session. Other requests for incentives included food. One father suggested, *“They come with trainings only and we are hungry*. *For children*, *the program teaches us how to feed them but there is no food*. *It would have been better to gift us with porridge flour for children*, *milk or food*.*”* (*Father*, *IDI #1*, *Vihiga*) Participants recommended other food, livestock, or agricultural items (e.g., seeds, chickens) as well as other material items (e.g., t-shirts, toys for their children).

Male and female caregivers, as well as other stakeholders, noted that men were reluctant to engage in sessions alongside women due to cultural and gender norms. Several mothers and fathers suggested holding separate sessions by gender because, as one woman stated, *“There are those [men] who have a mentality that they can’t sit with women in the same forum*.*”* (*Female Caregiver*, *IDI #5*, *Nyamira*) Similarly, one ADS supervisor highlighted the importance of creating male-centric spaces, stating, *"We need to do more of community awareness*, *male involvement activities*, *like father-to-father*, *joining male groups*, *which they can easily resonate to and buy in the idea*, *and in such*, *they just share their male challenges man to man*, *which is easier for them as compared with man-to-woman talk*. *This is because of the male ego*.*" (ADS Supervisor*, *IDI #2)* Additionally, both a supervisor and a male caregiver proposed the idea of employing male ECD promoters to bolster male participation, as most ECD Promoters are currently women and male participants may feel more comfortable receiving guidance from someone of the same gender. The same ADS supervisor as above additionally suggested, *“Call them for purely men training*… *if they are mentored by a male champion*, *I think that would also make it easier for them to be involved*. *It is easier for a man to mentor a man because they understand their challenges easier*. *So*, *father-father mentorship programs can really boost their participation in the program*.*” (ADS Supervisor*, *IDI #2)*.

When asked what else can be done to encourage more male participation, two faith leaders proposed reaching out to fathers in settings where men already commonly congregate, such as football fields, church events (e.g., men’s groups), “chamas” (savings group meetings), “barazas” (community meetings), and funerals. For example, one faith leader mentioned already trying to engage fathers in some of these different contexts, *“When I go to church*, *I explain to fathers that this program is not for the women only*, *but there is a role that fathers play in raising the children*. *I meet the fathers when we go for home visits*, *Chief Barazas*, *and they are aware of this program*.*” (Faith leader*, *IDI #1*, *Vihiga)* One ADS supervisor shared how they are trying to engage men more through faith leaders at church, *“We are using the faith leaders more so when they are doing their pulpit services to talk to men*. *Maybe when they are having those groups meeting in their churches where we have groups for men*, *for the children and for the mothers*. *We also encourage them to use those group meetings to share with them about the MTM*.*” (ADS Supervisor*, *IDI #1)* One faith leader also noted how faith-based platforms alone may not be applicable for men who are not very religious and therefore other strategies may be needed to reach these men.

Another recommendation that was mentioned less commonly by male caregivers and other community stakeholders was that the program should leverage participating mothers to reach their partners and encourage them to attend. An ECD promoter stated, *“As you meet primary caregiver*, *you also urge her to share with secondary caregiver what lessons learned*. *She should persistently push for the father to be convinced to join the program*.*” (ECD Promoter*, *IDI #2*, *Vihiga)* In addition, the program should also have mothers demonstrate to their partners what they have learned to get them to attend. As stated by a male caregiver, *“If those mothers could attend and learn and apply what they learn*, *it can make their men join the sessions*. *She must take lead in training and convincing the husband*.*”* (*Male Caregiver*, *IDI #1*, *Nyamira*).

Finally, participants also recommended incorporating program session topics that were especially geared towards fathers to pique their interest and more intentionally target and transform men. For example, two faith leaders recommended trainings on cattle farming, explaining that, *“Many men are involved in diverse personal projects*, *so to attract them*, *you will need to integrate the trainings with other empowerment trainings like for example cattle farming can be integrated with the program to attract more men*.*”* (*Faith Leader*, *IDI #3*, *Nyamira*) One program supervisor similarly recommended establishing a male caregiver forum in the community to talk about a variety of topics including those related to father involvement: *“You can even call them for purely men training to let them understand gender equality*, *to let them understand their role as fathers… I believe if we have more community awareness or sensitization during the male forum*, *as they are talking about their other things*, *you can chip in with the aspect of gender equality and male involvement*, *and they will easily buy into the idea*.*”* (*ADS Supervisor*, *IDI #2*).

## Discussion

In this study, we explored community members’ perceptions about father involvement in a parenting intervention for families with young children in rural Western Kenya. Specifically, we examined the perceived barriers and facilitators to father involvement in the program, the initial impacts of the program when fathers were involved, and the suggested recommendations for better reaching and including fathers in the program. Through interviewing female caregivers, male caregivers, delivery agents, and program staff, we found that most program participants were mothers with few fathers present at the parenting group sessions or home visits. Even though the program strategy was designed primarily for female caregivers, stakeholders still noted a wide range of positive changes for fathers. Overall, our findings highlight missed opportunities but also the potential of father-inclusive parenting programs and suggest specific strategies for more effectively engaging fathers alongside mothers in future programs.

One major theme that we uncovered was how community members reiterated fathers’ roles as financial providers as most important and perceived opportunity costs to men’s participation in community-based parenting interventions. Even those exceptional fathers who attended sessions articulated the tensions between men’s financial responsibilities and childcare roles within the household. One of the top recommendations from stakeholders was to provide financial incentives to encourage fathers’ program participation. The desire to receive financial assistance has been similarly highlighted as a key concern across many types of caregiving interventions seeking to engage fathers [19–21]. One notable program in Tanzania integrated livelihood support alongside a community group parenting program and engaged both mothers and fathers [22]. The financial and livelihood components of this program may have been a driving factor for why how this intervention achieved relatively high participation rates of fathers and improved multiple parenting and child-related outcomes. These types of multicomponent interventions that recognize the gendered divisions and societal expectations in parenting, particularly around men’s responsibilities as financial providers of the family, are likely to be effective in attracting fathers to the program and encouraging men’s engagement in other nurturing care roles [3, 12].

Another prominent barrier to men’s engagement in MTM was gender and social norms. These restrictive norms ranged from the belief that childcare is a women’s responsibility to stigma around men attending community group meetings alongside women to learn about such topics. To overcome some of the gender-related implementation barriers, participants in our study suggested incorporating some men’s only group sessions within MTM as a way of reducing some of these initial barriers, building peer support, facilitating attitude changes among men. While there are only a handful of parenting programs that have been designed to be gender-transformative, these programs have consistently proven to be successful in engaging fathers and improving a wide range of outcomes for men, women, couples, and young children [7, 9, 23]. In particular, studies across LMICs have underscored how fathers’ engagement in stimulation and responsive caregiving can independently improve various ECD outcomes, including children’s cognitive development, socioemotional, and behavioral outcomes, over and above the known positive contributions of maternal caregiving [10, 24]. However, perceptions of fathers’ parenting roles in many LMICs settings, including in Western Kenya as reaffirmed in our study, are largely equated to financial support [25, 26]. Thus, explicitly addressing gender both in the curriculum content itself to promote gender equality in caregiving roles, and in the delivery strategy to engage men, can facilitate wider and sustained changes for children, families, and communities.

Despite these barriers, all stakeholders underscored the importance of engaging fathers in the MTM program. Stakeholders offered multiple suggestions for overcoming the barriers to father involvement, which included providing incentives, engaging male mentors and other influential members in men’s social networks as program delivery agents, and scheduling program sessions that accommodate around fathers’ schedules and preferences. Our findings reaffirm similar suggestions identified in other implementation research studies about increasing men’s engagement in child and family interventions [3, 12, 14]. For example, in a process evaluation of a fatherhood program in Uganda, researchers found that strategic scheduling of group sessions was a crucial factor to ensuring high program attendance among fathers [15]. Other evaluations of successful father-inclusive interventions found that engaging trusted individuals to men–such as villages leaders or mentor fathers from the community–increased men’s buy-in and openness to the program [27, 28]. Future programs should conduct formative research at the outset of the project to identify the most promising implementation strategies and potential programmatic levers for engaging fathers vis-à-vis the barriers and facilitators in the given community context [29, 30].

### Limitations

There are several limitations to our study that are worth nothing. While we used a stratified sampling approach to increase variation, our study sample was limited to four communities, which may not capture the full range of implementation experiences pertaining to father involvement across all MTM communities. The majority of our sample (e.g., mothers, delivery agents, program staff) reported their perceptions about their male partners or fathers more broadly in the community, and we had a relatively small sample of fathers themselves. Future studies should include more male caregivers to capture a fuller understanding about fathers’ programmatic experiences and perceptions. Finally, the timing of this process evaluation was conducted during the first quarter of MTM to explore the quality of initial program implementation. Consequently, our results do not represent a complete account of fathers’ engagement in the MTM program as findings could potentially change by the end of the full MTM program cycle. Future research should build upon these findings by evaluating fathers’ program participation and the effects on fathers’ outcomes after the completion of the program and using mixed-methods.

## Conclusions

This study highlights the potential for engaging fathers in parenting programs. Stakeholders identified several barriers to fathers’ program participation such as men’s primary roles as financial providers, the lack of financial incentives, and restrictive gender norms that stigmatize male involvement in childcare activities. Nevertheless, they proposed multiple strategies for overcoming these barriers such as providing financial incentives, incorporating flexible scheduling, and engaging male champions in the community. Overall, this study emphasizes the importance of understanding male caregivers’ perspectives and adapting programs accordingly so they can be more inclusive and effective with fathers. Designing parenting programs that intentionally engage and support both mothers and fathers will likely maximize family participation, caregiving dynamics, and impact on early childhood development.
